# Piperlongumine induces apoptosis and reduces bortezomib resistance by inhibiting STAT3 in multiple myeloma cells

**DOI:** 10.18632/oncotarget.11988

**Published:** 2016-09-13

**Authors:** Yao Yao, Yueyue Sun, Min Shi, Dandan Xia, Kai Zhao, Lingyu Zeng, Ruosi Yao, Ying Zhang, Zhenyu Li, Mingshan Niu, Kailin Xu

**Affiliations:** ^1^ Blood Diseases Institute, Xuzhou Medical College, Xuzhou, Jiangsu, China; ^2^ Department of Hematology, The Affiliated Hospital of Xuzhou Medical College, Xuzhou, Jiangsu, China; ^3^ Laboratory of Pathology, Xuzhou Medical College, Xuzhou, Jiangsu, China; ^4^ Key Laboratory of Bone Marrow Stem Cell, Jiangsu Province, Xuzhou, China

**Keywords:** multiple myeloma, STAT3, piperlongumine, bortezomib resistance, immunoproteasome

## Abstract

Effective new therapies are urgently needed for the treatment of multiple myeloma (MM), an incurable hematological malignancy. In this study, we evaluated the effects of piperlongumine on MM cell proliferation both *in vivo* and *in vitro*. Piperlongumine inhibited the proliferation of MM cells by inducing cell apoptosis and blocking osteoclastogenesis. Notably, piperlongumine also reduced bortezomib resistance in MM cells. In a disseminated MM mouse model, piperlongumine prolonged the survival of tumor-bearing mice without causing any obvious toxicity. Mechanistically, piperlongumine inhibited the STAT3 signal pathway in MM cells by binding directly to the STAT3 Cys712 residue. These findings suggest that the clinical use of piperlongumine to overcome bortezomib resistance in MM should be evaluated.

## INTRODUCTION

Multiple myeloma (MM), a plasma cell disorder characterized by anemia, lytic bone disease, renal disease, and immune dysfunction, is the second most common hematologic malignancy [1, 2]. Many novel therapies, including proteasome inhibitors and immunomodulatory agents (IMiDs), have improved MM treatment during the past decade. The first-in-class proteasome inhibitor bortezomib is commonly used to treat MM. However, relapse following bortezomib treatment remains inevitable, and novel targets are needed for MM therapy [3–6].

Piperlongumine is an alkaloid isolated from the long pepper (*Piper longum L.*) that possesses anti-inflammatory, anti-platelet aggregation, and anti-tumor properties [7–9]. Piperlongumine exerts anti-tumor effects through a variety of pathways, including the induction of reactive oxygen species (ROS) accumulation, activation of C/EBP homologous protein (CHOP), suppression of LMP-1 (EBV-encoded oncogene) expression, activation of AMPK phosphorylation, inhibition of NF-κB, and promotion of autophagy [10–17]. Recently, Jarvius *et al.* found that piperlongumine inhibited the function of the ubiquitin-proteasome system (UPS) and subsequent ROS generation [18]. However, the mechanisms underlying these effects remain unclear.

Here, we investigated whether piperlongumine has anti-tumor activity in MM cells. We found that piperlongumine induced apoptosis not only in BTZ-sensitive, but also in BTZ-resistant, MM cells. Furthermore, we report for the first time that piperlongumine inhibited the activation of STAT3 by directly binding to Cys712 near the SH2 domain; piperlongumine had no anti-myeloma effects in cells with mutant STAT3 (C712A).

## RESULTS

### Piperlongumine inhibits proliferation and induces apoptosis in MM cells

**Figure 1:**
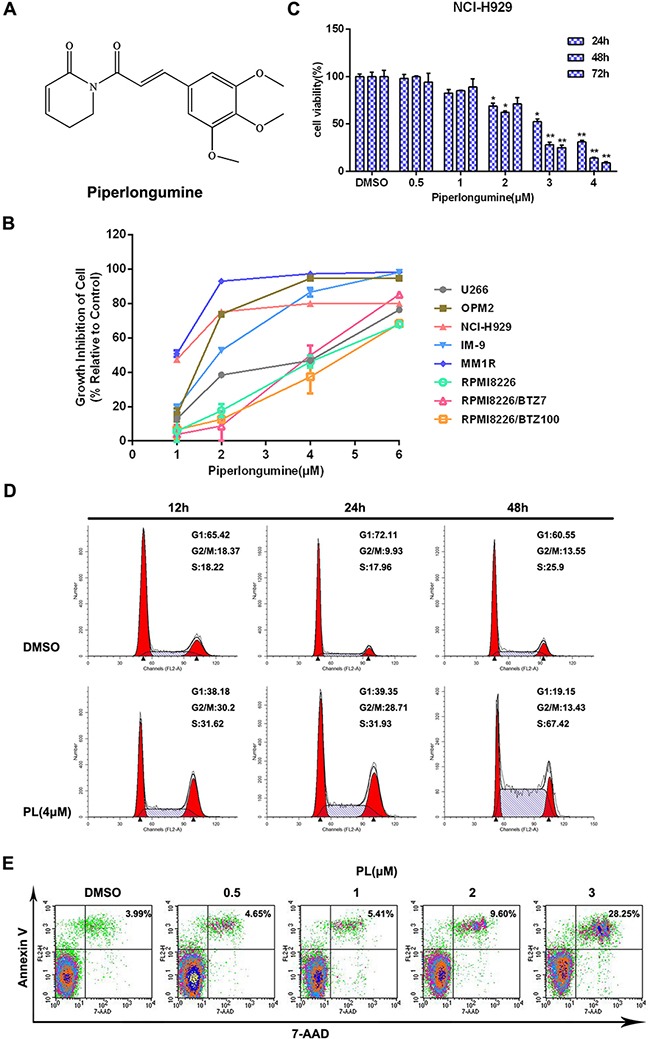
Piperlongumine inhibits cell proliferation and induces apoptosis in MM cells **A.** The structure of piperlongumine. **B.** Eight types of MM cells were treated with different concentrations of piperlongumine for 48 h and relative cell viabilities were then measured using a CCK-8 assay. **C.** Cell viability was measured in NCI-H929 cells treated with different doses of piperlongumine for 24, 48, or 72 h. **D.** After NCI-H929 cells were treated with 4 μM piperlongumine for 12, 24, or 48 h, relative numbers of cells in each cell cycle phase were analyzed by flow cytometry. **E.** NCI-H929 cells were treated with different concentrations of piperlongumine for 48 h and apoptosis rates were determined. All CCK-8 assay results were obtained from three independent experiments.

The effects of piperlongumine on the growth of various MM cell lines, including cells either sensitive or resistant to bortezomib (BTZ), were first determined by CCK-8 assay. Incubation with piperlongumine for 48 h inhibited MM cell growth in a dose-dependent manner, with IC_50_ values ranging from 1 to 5 μM (Figure 1B and Table 1). Treatment with different concentrations of piperlongumine for 24, 48, or 72 h also inhibited the growth of NCI-H929 cells in a dose- and time-dependent manner (Figure 1C). Similar results were obtained in IM9 and OPM2 cells (Supplementary Figure S1A); however, HS-5 stromal cells and normal hematopoietic cells were less sensitive to piperlongumine (Supplementary Figure S2).

**Table 1:** The IC50 values of seven human MM cell lines on 48h

Tumor cell line	IC50 (μM)
OPM2	2.7±0.5
MM1R	0.9±0.2
RPMI8226	2.8±0.8
NCI-H929	1.3±0.4
IM-9	2.1±0.2
U266	5.7±0.9
RPMI8226/BTZ7	3.3±0.8
RPMI8226/BTZ100	3.3±0.9

Cell cycle distribution and apoptosis rates were then examined in MM cells to investigate the mechanisms underlying piperlongumine's effects. Piperlongumine treatment increased the proportion of NCI-H929 cells in the S phase in a time-dependent manner (Figure 1D). In addition, piperlongumine increased apoptosis in a dose-dependent manner (Figure 1E). Similar cell cycle distribution and apoptosis results were obtained in IM9 and OPM2 cells (Supplementary Figure S1B-S1C).

### Piperlongumine induces MM cell apoptosis through both Fas- and mitochondria-dependent pathways

**Figure 2:**
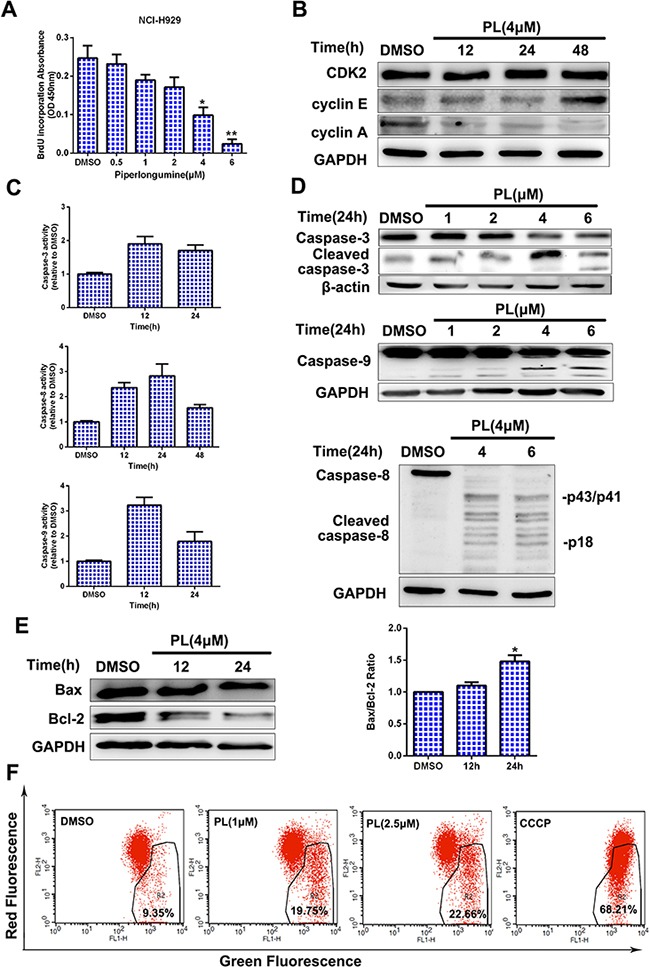
Piperlongumine inhibits DNA synthesis and stimulates apoptosis via Fas- and mitochondria-dependent pathways **A.** NCI-H929 cells were treated with piperlongumine (0.5, 1, 2, 4, or 6 μM) for 24 h, and a BrdU incorporation assay was then performed. Data are representative of three independent experiments. *P < 0.05, **P < 0.01, compared to control. **B-C.** NCI-H929 cells were treated with 4 μM piperlongumine for 12, 24, or 48 h; cyclins and CDK2 levels were then measured, and caspase activity was measured by colorimetric assay. **D.** NCI-H929 cells were treated with piperlongumine for 24 h, and cleaved caspase-3, caspase-9, and caspase-8 levels were measured. **E.** NCI-H929 cells were treated with 4 μM piperlongumine for 12 or 24 h and Bcl-2 and Bax levels were measured. Quantitative analysis was performed using Image J software, with normalization to GAPDH expression. **F.** NCI-H929 cells were treated with 1 or 2.5 μM piperlongumine for 12 h; CCCP was used as the positive control. Fluorescence was then measured by flow cytometry.

To determine how piperlongumine blocks cell cycle progression, a BrdU incorporation assay was performed to measure DNA synthesis. DNA synthesis decreased markedly in NCI-H929 cells after exposure to piperlongumine (Figure 2A). As expected, the expression of cyclin A, which promotes progression from the S to the G2 phase, decreased, while cyclin E expression increased; cyclin-dependent kinase (CDK) expression did not change (Figure 2B). Moreover, piperlongumine increased apoptosis in both time- and dose-dependent manners, as measured by caspase family (caspase-3, −9, or −8) cleavage and activity. As shown in Figure 2C and 2D, marked induction of caspase-3, −9, or −8 activity and cleavage were observed. Similar results were obtained in OPM2 and IM9 cells (Supplementary Figure S1D). Piperlongumine also decreased levels of the anti-apoptotic protein Bcl-2 and increased the Bax/Bcl-2 ratio in NCI-H929 cells (Figure 2E). In addition, piperlongumine treatment disrupted the mitochondrial membrane potential, as revealed by an increase in green fluorescence resulting from the cytosolic accumulation of monomeric JC-1 (Figure 2F). Intracellular ROS levels also increased after treatment (Supplementary Figure S1E). These data suggest that piperlongumine induces MM cell apoptosis through both Fas- and mitochondria-dependent pathways.

### Piperlongumine blocks osteoclastogenesis and cytokine secretion

**Figure 3:**
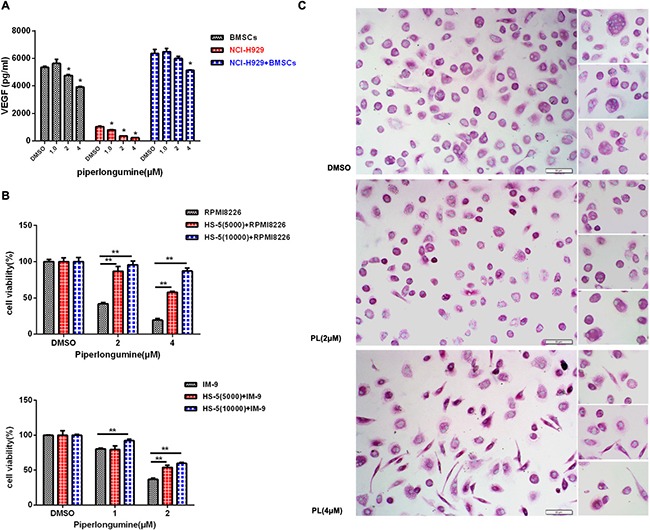
Piperlongumine targeted MM cells in the BM microenvironment and inhibited osteoclast formation **A.** NCI-H929 cells, cultured alone or with HS-5 cells, were treated with varying doses of piperlongumine, and conditioned media were collected for the measurement of VEGF levels using an ELISA. **B.** RPMI8226 or IM-9 cells were seeded into a 96-well microplate alone or with HS-5 cells, then treated with different concentrations of piperlongumine for 48 h and analyzed with a CCK-8 assay. Results are shown as the mean ± SD of three independent experiments. **C.** PBMCs isolated from normal donors (n=3) were incubated with piperlongumine, and the TRACP assay was performed to measure the formation of multinuclear osteoclast cells. The results are representative of three independent experiments.

Proliferation, survival, and avoidance of immune surveillance in MM cells all depend on the bone marrow (BM) microenvironment [19–21]. We therefore investigated the effects of piperlongumine on the BM microenvironment by measuring the secretion of VEGF from MM and BM stem cells, as well as osteoclast formation. As shown in Figure 3A, VEGF secretion decreased in NCI-H929 MM cells after piperlongumine treatment alone or together with co-cultured HS-5 cells (Figure 3A). MM cell growth also decreased after piperlongumine treatment with or without HS-5 cells (Figure 3B). Because osteolytic bone disease results from excessive osteoclast activation in most patients [22], an osteoclast formation assay was performed. As shown in Figure 3C, piperlongumine decreased numbers of TRACP-positive multinuclear cells in a dose-dependent manner. Together, these results indicate that piperlongumine may also inhibit MM cell growth and survival by altering the BM microenvironment.

### Piperlongumine inhibits the STAT3 signaling pathway in MM cells

**Figure 4:**
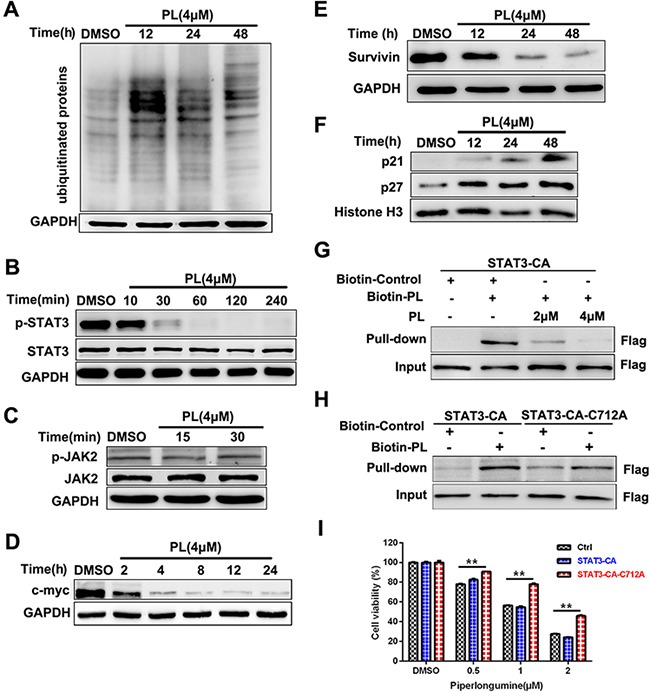
Piperlongumine inhibits STAT3 signal pathway activation **A.** NCI-H929 cells were treated with 4 μM piperlongumine, and poly-ubiquitinated protein levels were measured. **B-C.** NCI-H929 cells were treated with 4 μM piperlongumine for different amounts of time, and STAT3, p-STAT3, p-JAK2, and JAK2 levels were then measured. **D-F.** NCI-H929 cells were treated with 4 μM piperlongumine for different amounts of time, and c-myc, survivin, p21, and p27 levels were then measured. The data shown are representative of three independent experiments. **G.** 293T cells were transiently transfected with Flag-STAT3-CA plasmids and then treated with biotinylated piperlongumine (20μM) for 2 h in the presence or absence of pretreatment with unlabled piperlongumine for 1 h. The whole cell lysates were subjected to pull-down analysis with the use of streptavidin beads. Captured proteins were analyzed by Western blotting. **H.** 293T cells were transiently transfected with Flag-STAT3-CA or Flag-STAT3-CA-C712A plasmids and and then treated with biotinylated piperlongumine (20μM) for 2 h. The whole cell lysates were subjected to pull-down analysis with the use of streptavidin beads. Captured proteins were analyzed by Western blotting. **I.** NCI-H929 cells were infected with vector control, STAT3-CA, or STAT3-CA-C712A expression viruses, then treated with piperlongumine (0.5, 1, or 2 μM) for 24 h and analyzed using a CCK-8 assay.

To identify signal transduction pathways involved in the effects of piperlongumine, we measured activation of the NF-κB, MAPK, PI3K/AKT, UPS, and JAK/STAT3 pathways. Piperlongumine inhibited UPS function in NCI-H929 cells as demonstrated by the increased accumulation of poly-ubiquitinated proteins (Figure 4A). Furthermore, piperlongumine markedly inhibited STAT3 activity (Figure 4B), but did not affect the phosphorylation of JAK2, which acts upstream of STAT3 (Figure 4C). Next, we measured the levels of molecules downstream of STAT3, including c-myc, p21, p27, and survivin. Piperlongumine decreased survivin and c-myc levels and increased p21 and p27 levels (Figure 4D–4F). Activation of the NF-κB and MAPK signal pathways were not affected by piperlongumine, and PI3K/AKT activation was low in most MM cells (MM1R and JJN3) without cytokine treatment (IGF) (Supplementary Figure S3).

### Piperlongumine binds directly to STAT3 and inhibits its activity

To explore whether piperlongumine targets STAT3 directly, we performed a STAT3 pull-down assay using biotinylated piperlongumine. Treatment with biotin-piperlongumine resulted in STAT3 pull-down in 293T cell lysates, and treatment with unlabeled piperlongumine attenuated this binding (Figure 4G). This result and previous findings [23] indicate that piperlongumine may inhibit STAT3 activity by forming a covalent linkage with a specific cysteine residue. To confirm this, we mutated Cys712 to Ala; this mutation greatly reduced pull-down of STAT3 with biotin-piperlongumine (Figure 4G and 4H). We then developed cell lines that stably expressed either exogenous constitutively-active STAT3 (STAT3-CA) or C712A mutant STAT3As shown in Figure 4I, piperlongumine treatment inhibited growth in cells expressing vector or STAT3-CA; however, the anti-proliferative effects of piperlongumine were reduced in the cells expressing C712A mutant STAT3. These data suggest that piperlongumine inhibits STAT3 function by binding directly to the Cys712 residue.

### Piperlongumine reduces bortezomib resistance in MM cells

**Figure 5:**
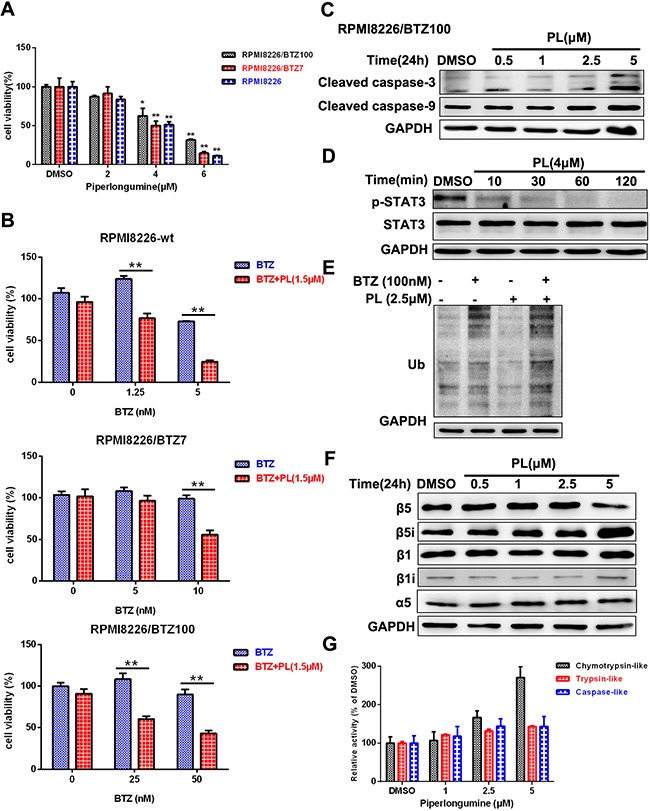
Piperlongumine inhibits viability in BTZ-resistant MM cells **A.** RPMI8226, RPMI8226/BTZ7, and RPMI8226/BTZ100 cells were treated with piperlongumine for 24 h and analyzed using a CCK-8 assay. **B.** Cells were seeded into a 96-well microplate, treated with piperlongumine (1.5 μM) for 12 h, and then treated with different concentrations of BTZ for 36 h and measured using a CCK-8 assay. **C.** RPMI8226/BTZ100 cells were treated with 0.5, 1, 2.5, or 5 μM piperlongumine for 24 h, and cleaved caspase-3 and caspase-9 levels were detected. **D.** RPMI8226/BTZ100 cells were treated with piperlongumine (4 μM) for 10, 30, 60, or 120 min, and p-STAT3 and STAT3 levels were measured. **E.** RPMI8226/BTZ100 cells were exposed to piperlongumine (2.5 μM) for 12 h followed by treatment with BTZ for 24 h, and poly-ubiquitinated protein levels were measured. **F.** RPMI8226/BTZ100 cells were treated with 0.5, 1, 2.5, or 5 μM piperlongumine for 24 h, and β1, β1i, β5, β5i, and α5 levels were measured. The data shown are representative of three independent experiments. **G.** RPMI8226/BTZ100 cells were exposed to piperlongumine (1, 2.5, or 5 μM) for 24 h, and proteasome activities were measured. Error bars represent standard deviations of the mean determined in a representative experiment performed in triplicate, and the results are representative of three independent experiments.

To explore the ability of piperlongumine to overcome BTZ resistance, we replicated the above experiments in BTZ-resistant cells. Cytotoxicity increased in both BTZ-sensitive and BTZ-resistant cells after exposure to a combination of BTZ and piperlongumine (1.5μM) (Figure 5A and 5B). Piperlongumine reduced the viability of BTZ-resistant cells by promoting apoptosis and decreasing STAT3 phosphorylation (Figure 5C and 5D).

Next, we examined the accumulation of poly-ubiquitinated proteins in BTZ-resistant cells after pre-exposure to piperlongumine. Treatment with bortezomib or piperlongumine reduced the accumulation of poly-ubiquitinated proteins. In contrast, pre-exposure to piperlongumine in combination with BTZ greatly increased the accumulation of poly-ubiquitinated proteins (Figure 5E and Supplementary Figure S4). β5i expression was increased, while β5 subunit expression was slightly decreased; however, β1 and β1i (LMP2) levels were unchanged (Figure 5F and Supplementary Figure S4). Proteasome catalytic activities, especially chymotrypsin-like activity, were increased by piperlongumine (Figure 5G and Supplementary Figure S4) [24]. Taken together, these results suggest that piperlongumine also exerts cytotoxic effects in BTZ-resistant cells by triggering apoptosis and inhibiting the STAT3 signal pathway, and partially restores BTZ sensitivity by increasing β5i levels.

### Piperlongumine inhibits human MM cell growth *in vivo*

**Figure 6:**
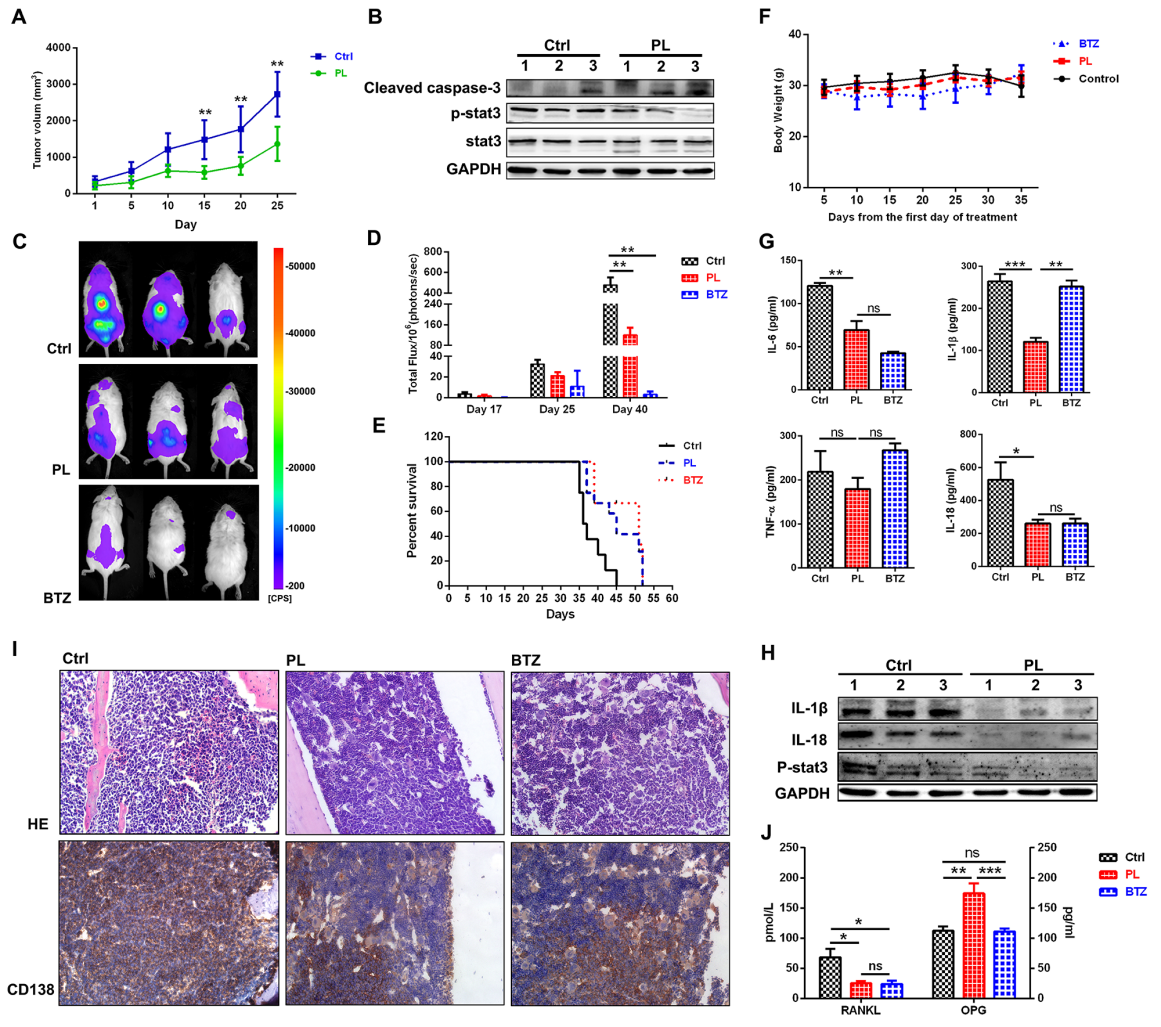
Piperlongumine exerted anti-MM effects and reduced MM-induced bone lesions in a disseminated murine model of human MM **A.** Nude mice were treated with vehicle (n=6) or piperlongumine (50 mg/kg, n=6) for 3 weeks. Piperlongumine treatment inhibited tumor growth compared to the control (*p*<0.01). **B.** WB analysis of mouse tumors showed that piperlongumine increased cleaved caspase-3 levels and decreased p-STAT3 levels. **C.** NSG mice were inoculated intravenously with RPMI8226-Fluc-GFP cells and then treated with vehicle (n=8), piperlongumine (50 mg/kg, n=12), or bortezomib (1.5 mg/kg, n=10) for 2 weeks. Tumor growth was measured by bioluminescence imaging (log scale). **D.** Bioluminescence imaging reported as mean photon flux (±SD). **E.** Log-rank analysis showed that median OS was prolonged in mice treated with piperlongumine (49 days) compared to the control group (39.5 days) (*P<*0.01). **F.** Treatment with piperlongumine or bortezomib did not significantly affect mouse body weights. **G.** Levels of secreted IL-6, IL-18, IL-1β, and TNF-α were measured in bone marrow cell supernatant using an ELISA. **H.** WB analysis showed that piperlongumine treatment reduced IL-1β and IL-18 levels in bone marrow cells compared to the control group. **I.** The extremities of the mice were stained with H&E and immunohistochemically analyzed for the indicated proteins. Original magnification, ×400. **J.** Levels of secreted RANKL and OPG in bone marrow cell supernatants were measured using an ELISA. ***P<0.001; **P<0.01; *P<0.05. ns: not significant.

We next examined the *in vivo* efficacy of piperlongumine using a human MM xenograft mouse model [25]. Mice treated with piperlongumine showed delayed tumor growth (*P*<0.01, Figure 6A). Western blotting analysis of tumors harvested from these mice indicated that cleaved caspase-3 levels increased, and p-STAT3 levels decreased, after piperlongumine treatment (Figure 6B). These results suggest that piperlongumine also exerts anti-MM activity *in vivo* in the plasmacytoma model.

The *in vivo* efficacy of piperlongumine was further evaluated in a disseminated MM model. Piperlongumine treatment alone markedly reduced tumor growth (Figure 6C and D, *P*<0.01) and survival (Figure 6E) compared to vehicle treatment. The mice in all three groups maintained similar body weights during treatment (Figure 6F). H&E and immunohistochemical staining were used to evaluate pathology in bone marrow isolated from the mice after drug treatment. As shown in Figure 6I, piperlongumine treatment decreased numbers of hemorrhages and infiltrated human myeloma cells compared to the control group. Bone lesions and pro-inflammatory cytokines were also evaluated in the disseminated MM model. Osteoclast (OC) hyperactivation results primarily from increased production of multiple “OC-activating factors” in the BM microenvironment (e.g., RNAKL, IL-6, IL-3, TNF-α, and IL-1β) [26, 27]. As shown in Figure 6G, H, and 6J, IL-6, IL-1β, RNAKL, and IL–18 levels decreased after piperlongumine treatment (*P*<0.05). In contrast, levels of osteoprotegerin (OPG), a decoy receptor for RANKL, increased in the piperlongumine group compared to the control and BTZ groups (*P*<0.01). Taken together, these data demonstrate that piperlongumine also exerted anti-MM effects and partially prevented bone resorption in a disseminated human MM model.

## DISCUSSION

In this study, we investigated the biological mechanisms by which piperlongumine exerts anti-cancer effects in human myeloma cells. We found that piperlongumine inhibits the proliferation of MM cells both *in vitro* and *in vivo* by inducing cell apoptosis. However, sensitivity to piperlongumine varies slightly in different MM cell lines, and especially in RPMI8226 and U266 cells. Consistent with previous reports, both of them are less sensitive to many anti-tumor agents than other MM cell lines [28, 29]. This may be due to the activity of multiple oncogenic signaling pathways that prevent apoptosis and increase drug resistance in RPMI8226 and U226 cells, Mechanistic studies have demonstrated that piperlongumine-induced apoptosis is associated with inhibition of the STAT3 signal pathway. Here, piperlongumine markedly decreased the expression of STAT3-regulated genes, such as VEGF, c-myc, Bcl-2, and survivin.

Piperlongumine has strong cytotoxicity in both BTZ-sensitive and BTZ-resistant MM cells, with no significant cross resistance. Our data indicate that this may be due to piperlongumine's ability to downregulate STAT3 signal pathway activity. The JAK-STAT3 signal pathway plays a critical role in the pathophysiology of MM, strongly promoting proliferation, survival, and drug resistance in myeloma cells [30]. The STAT3 protein is composed of 6 functional domains; the SH2 domain and the transactivation domain are especially critical for STAT3 phosphorylation, dimerization, and activation [23, 31]. Because the Cys712 residue is located near the SH2 domain, it is the critical site for covalent interactions between STAT3 and drug agents [23]. Here, we demonstrate for the first time that piperlongumine inhibits the STAT3 signal pathway in MM cells by directly binding to the Cys712 residue; a C712A mutation in STAT3 largely reversed the anti-myeloma effects of piperlongumine. Thus, piperlongumine may exert anti-myeloma effects by specifically inhibiting Cys712-dependent STAT3 activation.

Previous studies have demonstrated that levels of the constitutive proteasome subunit β5, which harbors mutations in the BTZ-binding pocket, are increased, and non-mutated immunoproteasome subunit levels are decreased, in BTZ-resistant cell lines [24, 32]. β5i and β1i expression are also down-regulated in BTZ-resistant MM cells, which may further contribute to resistance to BTZ-induced cytotoxicity. These data suggest that up-regulation of immunoproteasome expression may effectively reduce BTZ resistance in these cells [24]. Interestingly, we found that piperlongumine increased β5i expression and facilitated sensitization to BTZ. Furthermore, the immunoproteasome is slightly more efficient than the 20S proteasome in recognizing and degrading nascent oxidant-damaged proteins [33]. Recent studies have demonstrated that piperlongumine contributes to cancer cell death by targeting stress responses to ROS [9, 34–36]. Consistent with these studies, we found that piperlongumine increased intracellular ROS levels in MM cells. Therefore, we speculate that piperlongumine-induced oxidative stress may contribute to upregulation of the immunoproteasome (β5i), which in turn may increase selective degradation of oxidized proteins and help maintain protein homeostasis.

In summary, our results demonstrate that piperlongumine has strong anti-MM activity and inhibits STAT3 activation by binding directly to the Cys712 residue. Piperlongumine may therefore be a promising new agent for the clinical treatment of multiple myeloma, especially for bortezomib-resistant myeloma.

## MATERIALS AND METHODS

### Cell lines

OPM2, MM1R, U266, and IM-9 human multiple myeloma cell lines were obtained from the American Type Culture Collection (ATCC). The NCI-H929 cell line was obtained from the China Infrastructure of Cell Line Resources. The RPMI-8226 (BTZ IC_50_=2.6±0.3), RPMI8226/BTZ7 (IC_50_=12.1±0.7, resistance factor: 4.5), and RPMI8226/BTZ100 (IC_50_=105.9±14.9, resistance factor: 39.5) cell lines were kindly provided by Dr. J Cloos (VU University Medical Center) [24, 32]. All cells were cultured in RPMI 1640 (Gibco, USA) supplemented with 10% fetal bovine serum (Gibco, USA). The human stromal cell line HS-5 was also obtained from ATCC.

### Antibodies and reagents

Piperlongumine was purchased from Cayman Chemical (Ann Arbor, Michigan, USA). The apoptosis assay kit was purchased from eBioscience and the cell proliferation ELISA kit (BrdU) was purchased from Roche Diagnostics. Propidium iodide (PI), Ribonuclease A (RNase A), and dimethyl sulfoxide (DMSO) were purchased from Sigma-Aldrich (Shanghai, China). The cell counting kit-8 (CCK-8) was purchased from Dojindo Laboratories (Japan). Antibodies against p-STAT3, STAT3, PSMB6 (β1), PSMB8 (β5i), CDK2, cyclin E2, cyclin A, Bax, Bcl-2, c-myc, p21, p27, survivin, p65, p-p65, IL-1β, Histone-H3, caspase-8, cleaved caspase-9, and cleaved caspase-3 were purchased from Cell Signaling Technology (Beverly, MA, USA). PSMB5 (β5) and PSMB9 (β1i) were purchased from Abcam (Cambridge, MA). p38, p-p38, IL-18, ERK, p-ERK, and GAPDH were purchased from Santa Cruz Biotechnology, Inc. (USA). PSMA5 was purchased from Bioworld Technology. CD138 was purchased from Proteintech. Recombinant human macrophage colony-stimulating factor (M-CSF) and human soluble receptor activator of NF-κB ligand (sRANK Ligand) were purchased from PeproTech (Rocky Hill, NJ). STAT3-CA (constitutively active, A661C, N663C) plasmid was purchased from Addgene. The Cys712 of STAT3-CA was mutated to Ala (STAT3-CA-C712A) using the Fast Mutagenesis System.

### Cell proliferation assay

NCI-H929 cells (2 × 10^4^ cells/well) were seeded into 96-well plates and treated with different concentrations of piperlongumine. After 24 h, cells were labeled with BrdU (10 μM) for analysis of cell proliferation according to the manufacturer's protocol.

### Caspase activity assay

Caspase activity was detected using colorimetric assay kits (Bio-Box, China) according to the manufacturer's instructions. Briefly, 100-200 μg of cell lysates were added to a buffer containing a p-nitroaniline (pNA)-conjugated substrates for caspase-3 (Ac-DEVD-pNA), caspase-9 (Ac-LEHD-pNA), or caspase-8 (Ac-IETD-pNA) for analysis of caspase activity at 405 nm with a Multiskan FC Microplate Photometer (Thermo Scientific).

### Measurement of mitochondrial membrane potential

The JC-1 (5′, 6, 6′-tetrachloro-1, 1′, 3, 3′-tetraethylbenzimidazolylcarbocyanine iodide) Assay Kit (Life technologies, USA) was used to measure mitochondrial membrane potential disruption according to the manufacturer's protocol. Positive control cells were incubated with CCCP (carbonyl cyanide 3-chlorophenylhydrazone, 50 mM final concentration) for 5 min. Cells were washed once with PBS and analyzed using a flow cytometer.

### Measurement of pro-inflammatory cytokines

HS-5 (2×10^5^) cells were seeded into 24-well plates for 24 h to establish bone marrow stromal layers. NCI-H929 cells (2×10^5^) were added to the wells either alone or with the HS-5 stromal cell layer. Conditioned media was then harvested after 48 h of co-culture and secreted VEGF levels were detected using an enzyme-linked immunosorbent assay (ELISA) (MultiSciences). IL-1β, IL-18, IL-6, TNF-α, RANKL, and OPG levels in bone marrow cell supernatants were also measured by ELISA. The minimum detectable level for the cytokines was 10.0 pg/mL. All measurements were performed in triplicate.

### *In vitro* osteoclast culture and activity assay

Osteoclast (OC) differentiation from healthy donor peripheral blood mononuclear cells (PBMCs) was performed as previously described [37], [38]. The culture medium containing 25 ng/mL of M-CSF and 50 ng/mL of RANKL was refreshed three times a week, and piperlongumine was added at 2 or 4 μM. After 15 days of culture, cells were fixed and stained using a TRACP&ALP double-stain kit (TaKaRa Company) according to the manufacturer's instructions. TRACP+ OCs contain three or more nuclei per cell, and each OC formation assay was performed at least three times using PBMCs from different donors.

### Intact cell-based chymotrypsin-like, trypsin-like, and caspase-like proteasome activities

An intact cell-based Proteasome-Glo assay kit (Promega, Madison, WI) was used to measure basal and piperlongumine-induced proteasome activity according to the manufacturer's instructions. MM cells were exposed to piperlongumine for 24 h, and 20,000 cells were collected from each well for detection. After 10 min of incubation with an equal volume of Proteasome-Glo cell based reagent, luminescence was measured with a GloMAX 96 Microplate Luminometer (Promega).

### MM xenograft mouse model (plasmacytoma model)

To evaluate the anti-MM activity of piperlongumine *in vivo*, 5×10^6^ NCI-H929 cells were injected subcutaneously into the right flanks of nude mice (5-6 weeks old, female, Shanghai Slac Laboratory Animal Co. Ltd., Shanghai) [39]. Once tumors were measurable, mice received intraperitoneal injections of 50 mg/kg piperlongumine in 10% DMSO and 10% Tween 80 in water on 5 consecutive days a week for 3 weeks. The control group received vehicle injections under the same schedule. Tumor lengths and widths were measured every five days using calipers, and tumor volume was calculated with the formula V= π/6 (a×b^2^), where a and b are longer and shorter tumor diameters, respectively. Mice were killed when tumors reached 2cm^3^ or became ulcerated. Survival and tumor growth were evaluated from the first day of treatment until death. Tissue samples were collected, minced, and homogenized to extract whole cell lysates, and the clarified supernatants were used for Western blotting analyses.

### Disseminated MM model

To induce disseminated MM, male NOD-scid IL2rγnull (NSG) mice (Beijing Vitalstar Biotechnology Co., Ltd.) were inoculated intravenously with 5×10^6^ RPMI-8226-Fluc-GFP cells in 250 μL PBS and imaged five days later to determine baseline bioluminescence. Mice were divided into three groups with similar mean bioluminescence and received either 5 consecutive IP doses of vehicle (days 1-5), 50mg/kg piperlongumine (days 1-5), or 1.5 mg/kg bortezomib IP twice weekly (days 1 and 5), for 2 consecutive weeks. Bioluminescence imaging was performed weekly to monitor disease progression, and body weights were measured every 5 days.

### Bone marrow H&E and immunohistochemistry staining

Mice were sacrificed and femurs and tibia were isolated bilaterally, fixed with 4% paraformaldehyde solution for 48 h, decalcified with 10% EDTA for another 48 h, dehydrated, waxed, and sliced into 4 μm sections with an RM2126 microtome. After H&E staining, pathologic changes were evaluated using a light microscope. For immunohistochemistry, CD138 (1:100 dilution) antibody was added followed by incubation with HRP-conjugated secondary antibody. Sections were then prepared for and viewed using fluorescent microscopy.

### Statistical analysis

Data are shown as means ± SD or SE. Statistical significance between 2 treatment groups was analyzed using unpaired Student's*t*-tests with 2-tailed *P* values; statistical significance between multiple treatment groups was analyzed using one-way ANOVAs; statistical significance between multiple treatment groups over time was analyzed using 2-way ANOVAs; overall survival (OS) was measured using Kaplan-Meier survival analysis. All statistical analyses were conducted using GraphPad Prism software (version 6.0). *P* < 0.05 was considered statistically significant.

## SUPPLEMENTARY MATERIALS FIGURES



## Abbreviations

BTZ: bortezomib
PL: Piperlongumine
MM: multiple myeloma
BM: bone marrow
CDK: cyclin-dependent kinase
STAT3: signal transducer and activator of transcription 3
UPS: ubiquitin–proteasome system
ROS: reactive oxygen species
DMSO: dimethyl sulfoxide
ELISA: enzyme-linked immunosorbent assay
IFNγ: interferon γ
M-CSF: macrophage colony-stimulating factor
OC: osteoclast cell
sRANK: soluble receptor activator of NFκB
VEGF: vascular endothelial growth factor
