# Supplementary material for: Piperlongumine induces apoptosis and reduces bortezomib resistance by inhibiting STAT3 in multiple myeloma cells
Source: Oncotarget. 2016 Sep 13;7(45):73497–508. doi: 10.18632/oncotarget.11988 (PMC5341994; doi:10.18632/oncotarget.11988)
Supplement: Supplementary file 1 [file oncotarget-07-73497-s001.pdf]

Piperlongumine induces apoptosis and reduces bortezomib resistance by inhibiting STAT3 in multiple myeloma cells

SUPPLEMENTARY MATERIALS AND METHODS

Cell viability assay

These cells were cultured in 96-well plates followed by being treated with different concentrations of PL. Cell viability was determined by CCK8 assay.

Cell cycle analysis

These cells (1 × 10^6 cells/well) were seeded into 6-well plates and treated with 4 μM PL for 12, 24 and 48 h. Then, cells were harvested and measured as previously described. Cells were acquired by flow cytometry and cell cycle distribution was analyzed using ModFit LT 3.3 software.

Analysis of apoptosis

These cells (5 × 10^5 cells/well) were seeded into 24-well plates and treated with different concentrations of PL for 24 h. Then cell were harvested for measurement of apoptosis and detected according to the manufacturer's protocol.

Detection of the intracellular reactive oxygen species (ROS) generation

To assess the intracellular production of ROS, MM cells were treated with PL for different time, then cells were incubated in 5 μM 2'-,7'-dichloro-fluorescin diacetate (DCFH-DA, Beyotime Biotechnology, China) for 30 min at 37°C in dark. DCFH-DA was deacetylated into DCFH, which trapped in cell and oxidized by ROS to highly fluouescent compound, DCF (2',7'-dichlorofluorescein). Then cells were washed with PBS for twice, resuspended in 500 μl PBS, and detected using a flow cytometry.

Cell growth inhibition assay

According the previous study, prior to the experiment, BTZ-resistant cells were cultured in BTZ free medium for at least 4 days. Cells were preexposed for 48 h to 100 U/ml IFN-γ (PeproTech, Rocky Hill, NJ), then subjected to different concentrations of BTZ (5, 25, 50, 125, 250, 375 and 500 nM) for 48h and the cell growth inhibition was detected by CCK-8 assay.

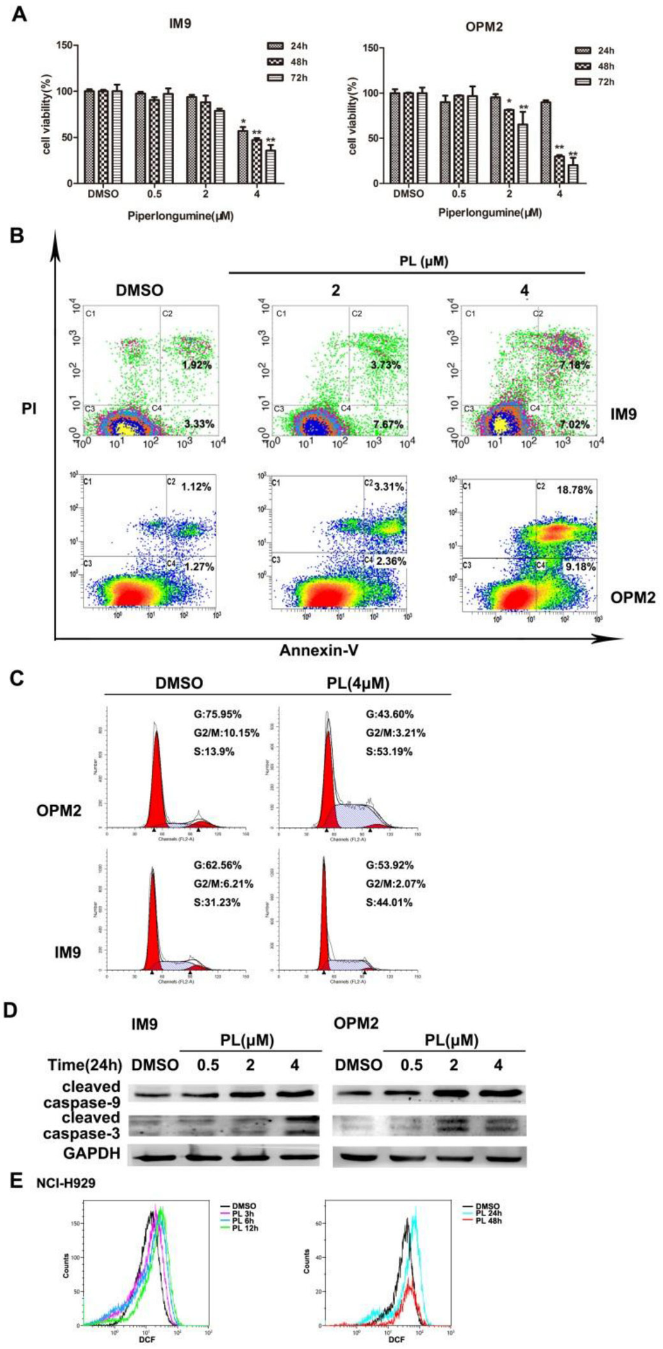

Supplementary Figure S1: PL blocks the cell cycle and triggers apoptosis in OPM2 and IM-9 cells. A. OPM2 or IM-9 cells were treated with DMSO or a dose range of PL (0.5, 2 and 4 μM) for 24, 48 or 72 h. Error bars represent standard deviations of the mean determined in a representative experiment performed in triplicate and all results of CCK-8 assays were performed three independent experiments. **B.** OPM2 or IM-9 cells were treated with DMSO or a dose range of PL (2 and 4 μM) for 24 h, and then the apoptotic rate were measured by flow cytometry. **C.** OPM2 or IM-9 cells were treated with DMSO or 4 μM PL for 24 h, then the relative number of cells within each cell cycle was determined by flow cytometry. **D.** OPM2 or IM-9 cells were treated with DMSO or a dose range of PL (0.5, 2 and 4 μM) for 24 h, the expression levels of cleaved-caspase3 or cleaved-caspase-9 were detected by western blot assay. Data shown are representative of three individual experiments. **E.** NCI-H929 cells were treated with DMSO or 2.5 μM PL for 3 h, 6 h and 12 h, or 24 h and 48 h, then the ROS accumulation was measured with DCFH-DA using a flow cytometer. The x-axis represents the fluorescence intensity and the y-axis represents the relative number of cells.

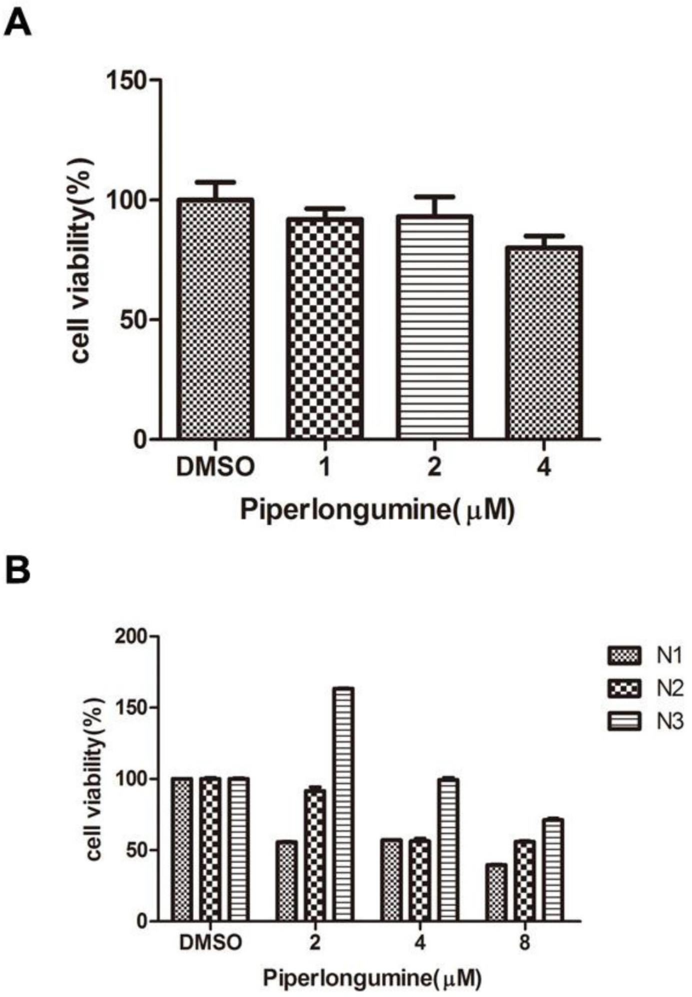

Supplementary Figure S2: PL has little cytotoxicity on HS-5 or normal hematopoietic cells. A. HS-5 cells were treated with DMSO or PL (1, 2 or 4 μM) for 48 h, and then cell viability were detected by CCK-8 assay. **B.** Normal hematopoietic cells from three healthy donors were treated with DMSO or PL (2, 4 or 8 μM) for 48 h, and then cell viability were detected by CCK-8 assay. Error bars represent standard deviations of the mean determined in a representative experiment performed in triplicate and all results of CCK-8 assays were performed three independent experiments.

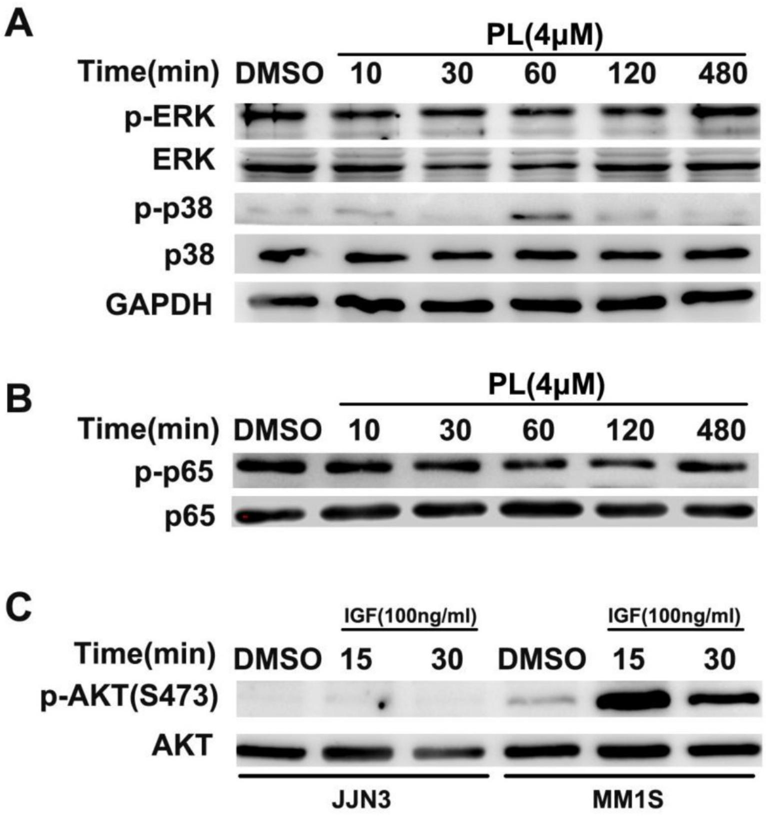

Supplementary Figure S3: PL has no effect on MAPK or NFκB signal transduction pathway. **A** or **B.** NCI-H929 cells were treated with 4 μM PL for 10, 30, 60, 120 or 480 min, then detected p-ERK, ERK, p-p38, p38, p-p65 or p65 using western blot assay. **C.** JJN3 or MM1S cells were preincubated with 100 ng/ml IGF for 15 or 30 min, then p-AKT was detected. Data shown are representative of three individual experiments.

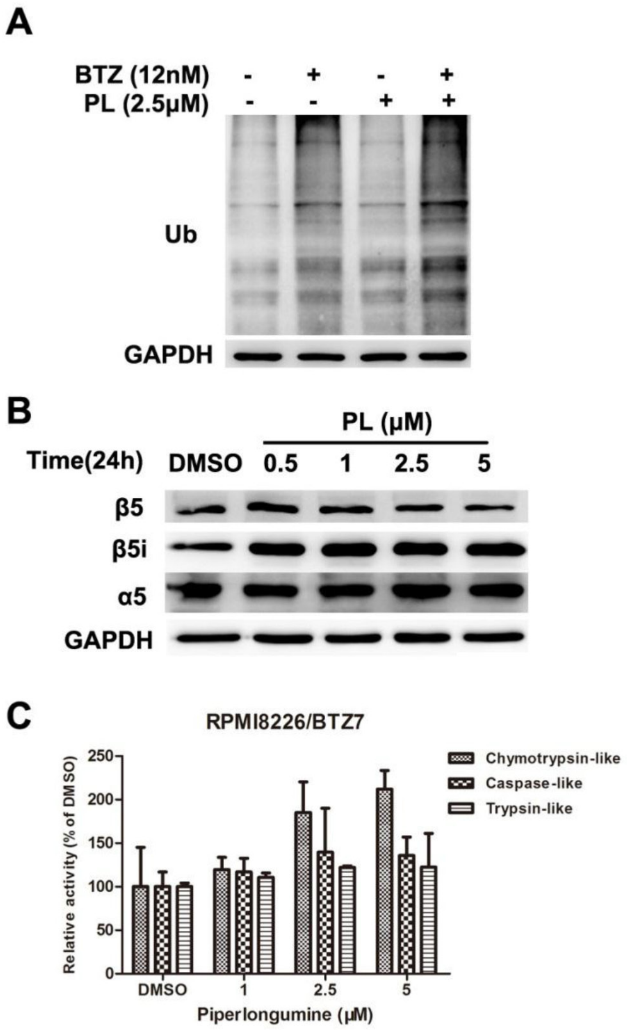

Supplementary Figure S4: PL's effect on RPMI8226/BTZ7 cells. **A.** RPMI8226/BTZ7 cells were exposed to PL (2.5 μM) for 12 h, then treated with BTZ for 24 h and the poly-ubiquitinated proteins were measured. **B.** RPMI8226/BTZ7 cells were treated with 0.5, 1, 2.5 and 5 μM for 24 h, and the expression of β5, β5i and α5 was detected. Data shown are representative of three individual experiments. **C.** RPMI8226/BTZ7 cells were exposed to PL (1, 2.5 or 5 μM) for 24 h, the chymotrypsin-like, trypsin-like and caspase-like proteasome activities were measured. Error bars represent standard deviations of the mean determined in a representative experiment performed in triplicate and the results are representative of three independent experiments.
